# Multivariate analysis of cytokine profiles in pregnancy complications

**DOI:** 10.1111/aji.12818

**Published:** 2018-02-15

**Authors:** Fawaz Azizieh, Kamaludin Dingle, Raj Raghupathy, Kjell Johnson, Jacob VanderPlas, Ali Ansari

**Affiliations:** ^1^ Department of Mathematics and Natural Sciences International Centre for Applied Mathematics and Computational Bioengineering Gulf University for Science and Technology Kuwait Kuwait; ^2^ Department of Microbiology Faculty of Medicine Kuwait University Kuwait Kuwait; ^3^ Arbor Analytics Ann Arbor MI USA; ^4^ eScience Institute Washington University Seattle WA USA

**Keywords:** cytokines, multivariate analysis, pregnancy complications

## Abstract

**Problem:**

The immunoregulation to tolerate the semiallogeneic fetus during pregnancy includes a harmonious dynamic balance between anti‐ and pro‐inflammatory cytokines. Several earlier studies reported significantly different levels and/or ratios of several cytokines in complicated pregnancy as compared to normal pregnancy. However, as cytokines operate in networks with potentially complex interactions, it is also interesting to compare groups with multi‐cytokine data sets, with multivariate analysis. Such analysis will further examine how great the differences are, and which cytokines are more different than others.

**Methods:**

Various multivariate statistical tools, such as Cramer test, classification and regression trees, partial least squares regression figures, 2‐dimensional Kolmogorov‐Smirmov test, principal component analysis and gap statistic, were used to compare cytokine data of normal vs anomalous groups of different pregnancy complications.

**Results:**

Multivariate analysis assisted in examining if the groups were different, how strongly they differed, in what ways they differed and further reported evidence for subgroups in 1 group (pregnancy‐induced hypertension), possibly indicating multiple causes for the complication.

**Conclusion:**

This work contributes to a better understanding of cytokines interaction and may have important implications on targeting cytokine balance modulation or design of future medications or interventions that best direct management or prevention from an immunological approach.

## 1 INTRODUCTION

Pregnancy is a natural phenomenon that has been a conundrum in immunology as the success of the allogeneic fetus seems to defy the rules of immunology. During pregnancy, the mother's immune system recognizes her fetus as “foreign,” yet instead of treating it as a target for rejection, the fetus is tolerated and nurtured through gestation. Indeed, pregnancy is a tight balancing act between different immune cells, hormones, nutrition, and infection under very strict immune regulation.^1^

This paradoxical success of the “fetal allograft” in the face of a potentially hostile maternal immune system has been suggested to be due to immunomodulation at the maternal‐fetal interface^2^, ^3^ and a consequent lack of strong maternal cell‐mediated anti‐fetal reactivity of the T helper 1 (Th1) type.^4^, ^5^ Th1 and Th2 cells represent two polarized forms of Th cells, and as the major functional subsets of Th cells, they mobilize different types of effector responses.^6^, ^7^ Th1 cells produce interleukin‐2 (IL‐2), interferon‐gamma (IFN‐γ) and tumour necrosis‐alpha (TNF‐α), induce several cytotoxic and inflammatory reactions and are responsible for cell‐mediated inflammatory reactions, delayed‐type hypersensitivity (DTH) and tissue injury in infectious and autoimmune diseases. On the other hand, Th2 cells secrete IL‐4, IL‐5, IL‐6, IL‐9, IL‐l0, and IL‐13 and are associated with help for antibody production by B cells.

It has been proposed that successful pregnancy in mice is a Th2 phenomenon and that abnormally elevated concentrations of Th1‐type cytokines are associated with spontaneous miscarriage in mice and humans.^8^, ^9^ It appears therefore that cytokines may have positive and negative effects on pregnancy depending on the types and levels of cytokines secreted.

Given this scenario, we and others have undertaken studies with the objective of elucidating the possible roles of cytokines in human pregnancy and to ascertain whether there are differences in cytokine profiles in normal human pregnancy as compared to unexplained pregnancy complications. We analysed supernatants of mitogen‐stimulated peripheral blood lymphocytes cultures, for a selected panel of Th1 and Th2 cytokines. Our data, as well as of others, support the hypothesis that normal successful pregnancy is a Th2‐phenomenon, while several other unexplained pregnancy complications such as recurrent spontaneous miscarriage (RSM), premature rupture of membranes (PROM), pregnancy‐induced hypertension (PIH), and preterm delivery (PTD) are associated with an elevated Th1‐type cytokine profile.^10^, ^11^, ^12^, ^13^, ^14^, ^15^, ^16^, ^17^, ^18^

However, the majority of the studies have compared individual levels or ratios of a small number of cytokines. Given that cytokines form a network of interacting entities and that a single cytokine or a ratio of two may not provide sufficient information about the overall immune reactivity, it is of interest to study the combined levels of several cytokines as this may provide a better picture of immune reactivity. Moreover, multivariate cytokine profile analysis may also suggest cytokine importance or a mathematical measure of the contribution of individual cytokines in separating the test group from its comparable/matching healthy control. This will shed light on the extent to which cytokine profiles are related (or can predict) different pregnancy conditions: If the cytokine levels can predict with high accuracy the pregnancy conditions, this would suggest that the cytokines are an important element of the disease process. If instead the cytokine levels only poorly predict pregnancy conditions, this would suggest that factors other than cytokines are contributing to a greater extent than just the cytokines being studied. This may also have important implications on targeting cytokine balance modulation or design of future medications or interventions that best direct management or prevention from an immunological approach.

Looking to pregnancy complication data from another angle, it does not appear that earlier work has focused strongly on *how different* these groups are. Rather, many previous studies have focused on whether statistically significant differences between groups of normal vs complication groups could be found by, for example, comparing the mean cytokine concentration values of each group. However, merely statistical significance via finding a small *P*‐value is not the whole story; it is also very interesting to know if the values are really very different, or only slightly different, despite having small *P*‐values. Indeed, we should consider the *practical* significance, not just the *statistical* significance, of the difference in cytokine values between groups. Practical, or clinical, significance can be expected to be related to the actual size of the difference; if the difference is small (but statistically significant), this suggests little practical or clinical significance or benefit. On the other hand, if the difference is large, then it suggests practical significance, such as the ability of a drug to alleviate symptoms.

Another question, which has received little if any attention in the analysis of cytokine data for pregnancy complications, is the presence of subgroups in the data. It may be helpful to investigate whether there are subgroups within the same pregnancy complication. Because the cause of the pregnancy complications studied was unknown (cases with known cause were excluded), patient groups may be made up of different subgroups within a certain complication. For example, cytokine levels may display a large difference from the normal pattern in 1 subgroup, while there may also be another subgroup of women in the same group presenting the same complication, but due to other unknown reasons where cytokines are contributors but not necessarily the major players.

Keeping the above points in mind, we aimed to use the statistical approaches to study and quantify the connection between cytokine profiles and different categories of pregnancy complications as compared to normal controls. More specifically, using the cytokine data we have previously gathered, here we investigate whether groups are different; how different they are; and in what ways they are different. Lastly, we also investigate whether there are subgroups within each complicated pregnancy condition.

## 2 MATERIALS AND METHODS

### 2.1 Subjects

The groups of subjects studied are as detailed elsewhere^10^, ^11^, ^12^, ^13^, ^14^, ^15^ and summarized in Table 1. The normal pregnancy control group consisted of women who were pregnant at the time of the study by spontaneous fertilization with a single fetus and had previously had at least 3 normal pregnancies, with no history of abortion, ectopic pregnancy, preterm delivery, or stillbirth and had normal spontaneous labor. All complication groups were properly selected according to the criteria set for defining the respective pregnancy complication and all subjects were investigated for possible anatomical, endocrinological, infectious, genetic, and immunological causes of the complication. These subjects had no demonstrable cause of their complication and therefore denoted as the unexplained cause.

**Table 1:** Groups of women studied along with the number of patients (n), clinical history, and mean gestational age

Group	Abbreviation	n	History	Mean gestational age
1st trimester	1st Tri	24	Women with a history of 3 or more normal pregnancies	12 ± 2
2nd trimester	2nd Tri	20		21.5 ± 0.6
Normal delivery	ND	53		39.4 ± 1
Recurrent spontaneous miscarriage	RSM	35	Women currently undergoing at least a third unexplained miscarriage.	12 ± 3
Preterm delivery	PTD	30	Women in premature active labor	26.8 + 1.3
Pregnancy‐induced hypertension	PIH	32	Previously normotensive women who developed hypertension associated with proteinuria during pregnancy	39 ± 1.4
Premature rupture of fetal membranes	PROM	30	Women with spontaneous rupture of fetal membranes at term	39 ± 1.1

The RSM group was comprised of women admitted with spontaneous abortion for evacuation, who have had at least 3 previous unexplained miscarriages, no earlier live birth (primary RSM) and who had been investigated clinically. The PTD group comprised women who were admitted to the hospital with spontaneous preterm labor, with intact membranes. These patients were in active labor, with the cervix dilated more than 3 cm and they delivered prematurely (before 34 weeks of gestation). Intrauterine infection was ruled out in these patients by high vaginal swab culture, urine culture, complete blood count, total and differential WBC count, and estimation of levels of C‐reactive protein. However, amniocentesis to exclude intrauterine infection was not feasible. The PIH group comprised women who: (i) were normotensive before pregnancy and during the first 20 weeks of gestation, (ii) developed hypertension (blood pressure > 140/90 mm Hg on two or more occasions 6 hour apart) associated with proteinuria >300 mg per 24 hour, and (iii) established labor either by induction or spontaneous onset. The PROM groups comprised women who were admitted with rupture of the fetal membranes at term prior to the onset of regular uterine contractions. Each group was compared to the gestationally age‐matched healthy normal pregnancy control group (ie, 1^st^ Tri vs RSM; 2^nd^ Tri vs PTD; ND vs PIH; and ND vs PROM) (Table 1).

Blood samples were obtained at the time of normal delivery (ND), spontaneous miscarriage (RSM), or anomalous end of gestation with PTD, PIH, or PROM. Therefore cytokine profiles reflect the situation existing in the periphery at that point in time. All subjects were enrolled at 2 high‐risk pregnancy clinics at Kuwait Maternity Hospital; informed consent was obtained from all subjects. All studies had the approval of the Ethics Committees of the Faculty of Medicine at Kuwait University and of the Maternity Hospital, Kuwait.

### 2.2 Mitogen‐induced activation of PBMC

Peripheral blood from subjects in the groups of Normal Delivery, RSM, PTD, PIH, and PROM were stimulated with mitogen. Peripheral blood was obtained by venipuncture from the subjects and PBMC were separated by Ficoll‐Hypaque (Pharmacia Biotech, Sweden) density gradient centrifugation, suspended in RPMI medium (GIBCOBRL, U.S.A) containing 10% fetal calf serum, aliquoted into 96‐well tissue culture plates at a density of 10^5^ cells per well and then challenged with phytohaemagglutinin (PHA) (Sigma Chemicals, U.S.A) at a concentration of 5 μg/mL for a period of 96 hours. Supernatants were collected for cytokine estimation on day 4 and stored at −80°C.

### 2.3 ELISA for cytokines

Cytokine levels were determined by sandwich ELISA using kits obtained from Immunotech SA (France). The manufacturer's protocols were followed up for the assays. Samples were tested in duplicate and absorbance values measured in an ELISA Reader. Accurate sample concentrations of cytokines were determined by comparing their respective absorbancies with those obtained for the reference standards plotted on a standard curve using reference recombinant cytokines. The profile consisted of the Th1 (IL‐2, TNF‐α, and IFN‐γ) and Th2 (IL‐4, IL‐5, IL‐6, and IL‐10) cytokines. All standards, controls, and samples were measured in duplicates. The sensitivity of each of the assays were as follows: 5 pg/mL of IL‐2, 10 pg/mL of TNF‐α, 3 pg/mL of IFN‐γ, 5 pg/mL of IL‐4, 1 pg/mL of IL‐5, 3 pg/mL of IL‐6, and 5 pg/mL of IL‐10.

### 2.4 Statistical analysis

All zero/undetectable cytokine concentration values were replaced with the minimum detectable (ie, sensitivity) value, as is standard practice. The small fraction of missing cytokine values were replaced with the median values of the samples (which is a conservative technique, not biased towards specific characteristics of the data). All concentrations were log‐transformed because a log scale is a more natural scale to study cytokine concentrations on. Log‐transforming is also standard in the literature. After this, the data were centered (by subtracting the mean) and scaled (divided by the standard deviation).

Some of the cytokine values (log transformed and scaled) deviated strongly from normal distributions; hence, we employed non‐parametric statistical tests to measure the differences in groups (see below). A *P*‐value of <.05 was considered statistically significant in this study. Data and statistical analysis were performed with *Scikit‐learn* 19 using the *iPython interface*.^20^

We compared the multivariate cytokine data between groups. To do so, the main tools used were as follows:

1 Multivariate comparison of the cytokine pattern between each of the complication groups with its gestational age‐matched control using the **Cramer Test** 21 on RStudio.^22^ This non‐parametric test gives a *P*‐value from comparing 2 multivariate distributions, testing whether the 2 groups are different or not with respect to the pattern of *all* measured cytokines. Such multivariate tests have the advantage of being able to handle multiple variables, and their potentially complex interactions and correlations. On the other hand, as with all such multivariate tools, their ability to detect small univariate differences is slightly weakened, due to the “noise” introduced by potentially irrelevant variables.
2 To investigate **Cytokine importance**, ie, which cytokine variables are most responsible for separating 2 groups, we used the classification algorithm CART (classification and regression trees) where “importance” give the average weighting of each cytokine indicating how strongly each cytokine drives the separation between 2 groups. The method gives a list of the cytokines and a value from 0 to 1, indicating how important the cytokine is in separating the groups, where higher values indicate greater importance. The default gini method was used for creating trees. Hyper‐parameters were optimized by cross‐validation while searching over these values: Max tree depth 1, 2, 3, and 4; minimum samples per leaf 5, 6, 7; the number of features to consider when looking for the best split was 2, 3, 4,…, 7. Cross‐validation was undertaken by 500 random stratified samples, splitting the data into train/test sets. The stratified test sets were chosen to be 10% of the number of samples of data, as is standard practice. As a comment, it is true that studies of individual or ratios of cytokines can also give cytokine importance. For example, if a set of cytokines is compared between 2 groups, and a few are found to be statistically different, then these cytokines would, of course, be important in distinguishing the groups. However, the individual/ratio approach has a weakness in that it is not straightforward to say which cytokines are *more* important or to rank them in terms of importance. Such a ranking has obvious clinical relevance, for example, in drug design and treatments.
3 Evaluating *how* different the groups are, using various methods:
  - **Partial least squares regression (PLSR) figures**—This method will plot a 2D projection (or “shadow”) of the multivariate data of all cytokines tested. The algorithm searches for the best way to project the data into a lower dimension so as to identify the best linear separation between the groups. If the projections of each group overlap a lot, this suggests that the cytokines value distributions are quite similar; if they do not overlap a lot, it means that the characteristics of the cytokines are quite different between the groups. Such visualizations aid in highlighting in what ways the 2 groups differ, for example, whether 2 groups are completely separate, or 1 group is a subset within another group, etc. As a final cautionary comment, with a smaller number of samples and a larger number of variables, PLSR projections will tend to overemphasize the difference between 2 multivariate groups, because in the algorithm searches many projections for the best way to separate the 2 groups (Cf. “p‐hacking”). Hence, because some of our group samples are not large, the projection separations and K‐S values (below) may be a little larger than the true values (which might be obtained with very large samples).
  - **2‐dimensional Kolmogorov‐Smirmov test—**While PLSR visualizations are helpful, it can sometimes be hard to visually judge how much or how little overlap there is between groups. With this in mind**,** a more quantitative way to compare the projections of 2 groups is to use the PLSR 2 dimensions (2D) plots and compare the 2 groups using the 2D analog of the Kolmogorov‐Smirmov (K‐S) test.^23^ Similar to the K‐S test with univariate data, the K‐S test in 2D measures the distance difference between 2 distributions in 2D data. Hence, we can use it on the PLSR projections to quantitatively measure how different the 2 groups are. The difference between 2 groups in 2D ranges between 0 and 1, with higher values indicating larger differences.
  - **Classification ability—**Another way to measure how strongly 2 multivariate groups differ in distribution is to compare how accurately a classification algorithm can classify samples from each group. Intuitively, if 2 groups have very different cytokine values, then samples from each group will be easily classified, or associated, to the correct group. Conversely, if the cytokine profiles of 2 groups are very similar (or identical), then a classification algorithm will only be able to correctly classify a small fraction of the samples (or none at all). Here, we used the CART classification trees to perform such classification of train/test samples for each pair of groups (as described above). However, instead of measuring the classification accuracy, we report the ROC AUC: the mean receiver operator characteristic (ROC) area under the curve (AUC) for the predicted class of the test samples (during cross‐validation). The ROC AUC is a standard method for measuring the accuracy of binary classification predictions. The AUC score will be 0.0 if all the predictions are incorrect, and a score of ~0.5 corresponds to just random guessing (ie, very poor) classifications, and a score of 1.0 corresponds to perfect classification accuracy. Hence, for example, a score 0.6 indicates a fairly poor classification ability, while 0.9 is very good. The reason for using the AUC instead of simply the classification accuracy (fraction of correct classifications) is that the relative proportion of each class largely determines the accuracy. So, for example, if 90% of the samples are 1 class and the other 10%, then a 90% accuracy could be achieved even by an algorithm that assigns all sample to large class. Hence quoting the accuracy can be misleading indicator of classification ability, while the ROC AUC accounts for different class proportions.
4 Finally, we investigated if there were subgroups within each complication group. To do this, we first used **principal component analysis (PCA)** to visualize the multivariate data in 2 dimensions. Doing so helps to reveal any obvious clustering or grouping. We complemented this method using the **gap statistic** (using **k‐means clustering** *),* 24 which is a standard method for finding the number of subgroups in multivariate data, and identifying subgroup membership for each sample. Searching for subgroups in relatively small multivariate data samples can be challenging, so we may not be able to detect subgroups which are present if there is only weak clustering of data. Nonetheless, we can expect to find subgroups if there is strong clustering.

## 3 RESULTS

### 3.1 Multivariate comparison of the cytokine pattern between groups

The multivariate Cramer Test detects more complex differences in cytokine patterns as opposed to single or simple ratios differences between groups. As shown in Table 2, cytokine patterns in pregnancy complications were statistically significantly different as compared to the respective groups of matching gestational age. This indicates the statistical significant differences in cytokine profiles between healthy and complicated pregnancy groups of RSM, PTD, PIH, and PROM (*P* = .0001, .023, .0097, and .009 respectively) (Table 2).

**Table 2:** Results of multivariate Cramer test, cytokine importance and classification accuracy for all combinations

Groups	Multivariate *P*‐value	Cytokine importance	K‐S distance	ROC AUC
**1**st **Tri vs RSM**	.0001	IL‐10 1.0	0.92	0.88
**2**nd **Tri vs PTD**	.023	IL‐2 0.53 IFN‐γ 0.32	0.58	0.78
**ND vs PIH**	.0097	IL‐10 0.33 IL‐5 0.17	0.53	0.74
**ND vs PROM**	.009	IL‐5 0.68 IL‐4 0.25	0.57	0.68

K‐S: 2‐dimensional Kolmogorov‐Smirnov distance.

ROC AUC: receiver operator characteristic area under the curve.

### 3.2 Cytokine importance using the classification algorithm

Table 2 further lists cytokines that most contributed to the multivariate differences and allocates an importance value for each. The table lists all importance that are more than 0.1 (ie, more than 10% contribution).

The classification algorithm related the significant multivariate difference between RSM and healthy gestational age‐matched controls mainly to IL‐10. With respect to 2nd Tri vs PTD, maximum cytokine importance were for IL‐2 (0.53) and IFN‐γ (0.32). As compared to ND, cytokine importance for PIH were those of IL‐10 (0.33) and IL‐5 (0.17), while those for PROM group were IL‐5 (0.68) and IL‐4 (0.25) (Table 2).

### 3.3 Evaluating how different the groups are, using various methods

**Figure 1:**
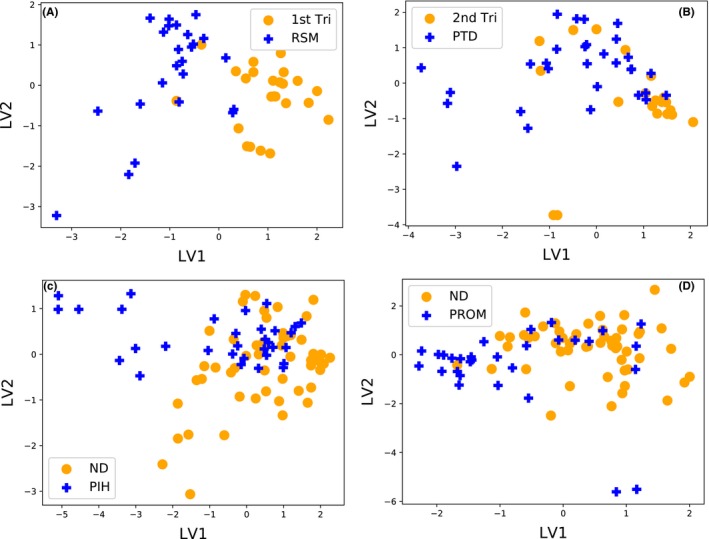
Visualizing multivariate cytokine profiles in 2 dimensions with PLSR projections. (A) 1st Tri vs RSM; (B) 2nd Tri vs PTD; (C) ND vs PIH; (D) ND vs PROM. The x and y axes are *latent variables* 1 and 2. These axes are chosen to maximize the separation between the two groups. Strongly overlapping groups suggest the cytokine profiles of the two groups are very similar, while clear separation between groups implies that the cytokine profiles are very different

To understand better how strongly any 2 groups differ from each other, and in what way they differ, we plotted the PLSR projections (Figure 1) reported the 2‐dimensional Kolmogorov‐Smirmov distance and calculated the classification accuracy as compared to the ROC AUC (Table 2).

Summarizing the information on all these tests for all groups is as follows:

Among the tested groups, 1st Tri and RSM are the most separated, which we infer from the obvious separation between the groups in the PLSR figure (Figure 1A), the large K‐S distance (0.92), and a high ROC AUC (0.88) (Table 2).

2nd Tri is also different from PTD and appears more clustered on the edge of the PTD projection (Figure 1B). Visually, the 2 groups are quite different, but also have some points with overlap. Further, the K‐S distance of 0.58 and ROC AUC value of 0.78 suggest that the 2 groups are different, but not extremely distinct, and certainly less than the separation between 1st Tri and RSM groups (Table 2).

Although the ROC AUC between ND and PIH is somewhat high (0.74), they show considerable overlap in cytokine levels in PLSR figure (Figure 1C). Further, the K‐S distance is lowest among other comparisons (0.53) pointing that the groups are somewhat different, but not strongly distinct (Table 2).

Finally, comparing ND to PROM, Figure 1D shows quite a strong overlap between the groups, suggesting that the cytokine profiles are quite similar. The K‐S distance is quite low (0.57), and the ROC AUC value also points to the groups being only modestly different (0.68) (Table 2).

Thus we see that while all pairs of groups are statistically significantly different with small *P*‐values in multivariate Cramer test, they vary considerably in both how strongly, and in which ways they differ.

### 3.4 Investigating subgroups within complication groups

We further investigated whether there exist subgroups within each of the pregnancy complications (RSM, PTD, PIH, and PROM). The reason for this investigation is that subgroups may imply different causes for the complication.

**Figure 2:**
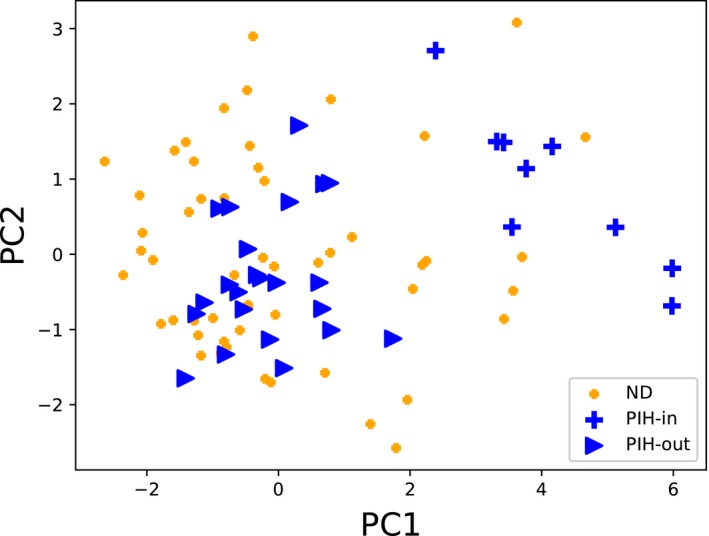
A PCA plot of ND data (yellow dots), with the PIH data (blue crosses and triangles) projected onto the same axes. The crosses and the triangles denote the 2 subgroups found by visual inspection, the gap statistic, and k‐means clustering

We were not able to find clear evidence of subgroups within RSM, PTD, or PROM. However, the clearest result of clusters among a pregnancy complication was in the PIH group. To search for subgroups, we first examined PCA plots of all the groups. Figure 2 shows the PCA projection of the PIH data onto the first two principal components of the ND data. Visually examining the PCA plot in Figure 2 suggests that the PIH group is made up of 2 subgroups, which we have shown as crosses and triangles. We will call the PIH subgroup on the “edge” of the PCA projection *PIH‐out*, and the other group *PIH‐in*. Using the Gap Statistic, we also found that the PIH group has 2 subgroups/clusters. Further, employing the K‐means clustering method (with K = 2) to assign each sample to 1 of 2 subgroups, we find an exact agreement between the subgroups visually apparent in the PCA plot, and the subgroups found by the gap statistic with K‐means.

**Figure 3:**
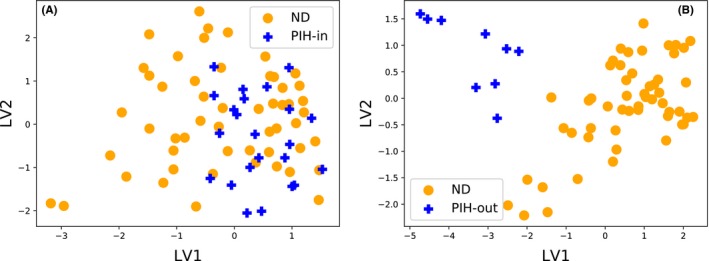
Visualizing multivariate cytokine profiles in 2 dimensions with PLSR projections for ND vs. PIH subgroups. (A) ND vs the PIH‐in; (B) ND vs PIH‐out. The x and y axes are *latent variables* 1 and 2. These axes are chosen maximize separation between the two groups. Strongly overlapping groups imply the cytokine profiles of the two groups are very similar, while clear separation between groups implies that the cytokine profiles are very different

Having found 2 subgroups, it is interesting to analyze them further. Figure 3 presents the PLSR projection for each of the subgroups as compared to ND. Among the 32 PIH patients points, 23 appear to have cytokine profiles which are typical of ND patients (denoted as PIH‐in, Figure 3A), while the remaining 9 form a subgroup which has cytokine profiles that are quite different to ND (denoted as PIH‐out, Figure 3B).

Table 3 further depicts the analysis of the 2 subgroups as compared to the ND group. As expected, the multivariate Cramer Test *P*‐value, the K‐S distance as well as the classification accuracy are far more distinct between ND vs PIH‐out as opposed to ND vs PIH‐in combination. In other words, the subgroup PIH‐in is very similar to ND, but not exactly identical in distribution; while the subgroup PIH‐out is very different from ND.

**Table 3:** Results of multivariate Cramer test, cytokine importance, and classification accuracy for PIH subgroups

Groups	Multivariate *P*‐value	Cytokine Importance	K‐S distance	ROC AUC
ND vs PIH	.0097	IL‐10 0.33 IL‐5 0.17	0.53	0.74
ND vs PIH‐in	.087	IFN‐γ 0.54 IL‐10 0.19	0.47	0.65
ND vs PIH‐out	.0000001	IL‐5 0.89 IFN‐γ 0.10	1.0	0.91

K‐S: 2‐dimensional Kolmogorov‐Smirnov distance.

ROC AUC: receiver operator characteristic area under the curve.

In summary, we found that the PIH group is actually made up of 2 subgroups/clusters, one having a multivariate cytokine profile very similar to ND, while the other subgroup is quite different to ND. This suggests that among the patients in our PIH group, a subgroup (PIH‐out) had PIH consequences which were reflected/shown by the cytokine imbalances as compared to ND, while this was not the case for the PIH‐in subgroup of patients suggesting their presentation is not mediated/reflected by cytokine imbalances.

It is worth also noting that the multivariate Cramer Test that detects differences in cytokine patterns was not significantly different between ND and PIH‐in group (*P* = .087), while, as expected, the *P*‐value between ND and PIH‐out was extremely significant (*P* = .0000001). The maximum cytokine importance/contribution in PIH‐out group were for IL‐5 (0.89) and IFN‐γ (0.10), (Table 3). The large K‐S distance (1.0), and large ROC AUC value of 0.91 corroborate the same finding (Table 3).

## 4 DISCUSSION

Cytokines are known to work in a complex hierarchical network and most of them show pleiotropic, redundant, and synergetic actions, making the full understanding of the balance very challenging. While several reports have suggested the use of cytokines as potential biomarkers, a single biomarker may be insufficient; thus it was suggested that it would be more appropriate to use ratios of 2 cytokines or to develop a multivariate “cytokine signature” based on the pattern of several cytokines produced by peripheral blood mononuclear cells.^25^, ^26^, ^27^

We compared multivariate cytokine profiles of several pregnancy complications to gestationally age‐matched control groups, using several statistical techniques. It was previously established that the groups are different in terms of individual cytokine levels and simple ratios, but we sought to investigate these differences further. Studying all tested cytokines, our main aim was to see how different are the panels and in which ways they are different in pathological pregnancies as compared to normal controls.

We, therefore, analyzed our data with a variety of multivariate statistical techniques that can detect more complex differences in patterns than simple differences in individual levels or ratios. It is useful to employ such multivariate methods because it is in principle possible that none of the individual variables show statistically significant differences in median levels when comparing samples of 2 groups, while a multivariate method may nonetheless uncover a difference in the multivariate pattern. On the other hand, the ability to detect multivariate differences comes at the expense of a reduced ability to detect individual cytokine differences.

The multivariate analysis showed significant differences between normal pregnancy and pathological complications. This may reflect the complexity and importance of cytokine balance in pregnancy and encourage further studies to select and include more parameters, study other complications and examine other disease conditions. Building on this, and considering the potential of the clinical approach, it would be very useful to know which of the tested cytokines most contribute to the deviation from the normal physiological cytokine balance. Using the data available, we used a classification algorithm (CART) and found the mean contribution of individual cytokines and their importance in differentiating the pathological presentation from the normal cohort.

It is interesting that the multivariate cytokine profiles of RSM and healthy controls were quite distinct and the main cytokine separating the 2 groups was IL‐10. IL‐10 has been shown to play a pro‐pregnancy role in early gestation,^28^, ^29^ essential for the implantation of the blastocyst and formation of the early placenta; it inhibits the secretion and synthesis of the deleterious pro‐inflammatory cytokines such as IL‐2, TNF‐α, and IFN‐γ by Th1 cells 30 and down‐regulates major histocompatibility complex (MHC) class II antigen expression.^31^ On the other hand, IL‐10 serves to modulate trophoblastic invasion, maintains an anti‐inflammatory milieu,^32^ stimulates placental angiogenesis^33^ and acts as a mediator of other intrauterine regulators such progesterone, catecholamines and prostaglandins.^29^

Similarly, both the PIH and PROM groups showed lowered Th2 cytokines to be the most differentiating cytokines as compared to normal delivery. IL‐4 and IL‐5 are classical Th2 cytokines and several researchers have reported the association of their lower levels with pregnancy complications.^15^, ^17^, ^34^, ^35^

However, the main cytokine importance for the PTD group were of the Th1 cytokines IL‐2 and IFN‐γ. These findings are in concordance with what has been reported by several investigators on the role of pro‐inflammatory cytokines in PTD. For example, it was demonstrated that the presence of IFN‐γ in cervicovaginal fluid in late second and early third trimesters is an important risk factor for PTD in asymptomatic women.^36^ Similarly, increased production of the pro‐inflammatory cytokines IL‐1, TNF‐α, and IL‐6 by placental cells and by amniotic and chorionic decidual tissues in PTD has been reported.^11^, ^37^

It is also interesting and novel that the analysis of our PIH group showed that it is made up of 2 clusters, one with multivariate cytokine profile, that is, similar to healthy controls, while the other is quite distinct. This method of analysis and the findings may help in explaining the long and wide controversy in literature about the association of different cytokines in different pregnancy complications. Taking PIH as an example, while there is substantial evidence supporting a role of cytokines in the pathogenesis of PIH, the underlying pathophysiologic mechanisms are still unclear with several proposed pathways.^35^ It is possible that among immune causes, different immune mechanisms operate at different interfaces during the different stages of pre‐eclampsia, where the final stage in all cases would be placental damage and the manifestations of PIH.^35^, ^38^ Taking into consideration other factors, such as HLA (Human Leukocyte Antigen) expression by the trophoblast, secreted trophoblast‐derived factors, cytokine genotyping polymorphism and others, all contribute to the complexity and warrant further consideration.^35^, ^38^, ^39^, ^40^

To the best of our knowledge, this is the first study to report simultaneous measurement of multiple cytokines from several different pregnancy complications followed by comparisons of cytokines in multivariate statistical pattern techniques as compared to healthy controls. However, there are limitations. From a biological point of view, measuring the production of certain cytokines by PBMC in an in vitro setup only partly reflects the much more complicated in vivo scenario at the feto‐maternal interface, where T‐cells are in close contact with other regulatory lymphocytes, natural killer cells and a multitude of interacting cytokines and factors. From a statistical point of view, the statistical power of multivariate analysis is dependent on sample numbers, and there is an increased risk of “over‐fitting” or observing false positive results when analyzing high‐dimensional data with just few samples (many cytokines, but few samples). Given our data set of a small number samples, and especially because the data has several variables ie, several cytokines, both the accuracies and cytokine importance values should be taken as tentative results, subject to future corroboration, or adjustment. Having said that, our analysis and findings do point to several interesting avenues to be explored further.

Several authors have modeled cytokine‐mediated inflammatory processes; examples include rheumatoid arthritis^41^ and cancers.^42^ Modeling and multivariate cytokine analysis may well prove to be valuable in devising strategies for immunomodulation in pregnancy complications. If studies demonstrate a linkage between Th1 cytokines and pregnancy failure, or if cytokine models can be developed to predict the profile of conditions; effort in the future could be directed at shifting the overall immune bias away from Th1‐dominance towards Th2‐bias (or whichever cytokine levels the model predicts is most appropriate). This may be attempted by down‐regulating Th1 cytokines or neutralizing them in such a manner as to allow pregnancy to proceed normally in a Th2‐biased milieu. This might well establish a basis for future therapeutic and prophylactic interventions in women with pregnancy complications and also pave the way for such manipulations in other immunological diseases.

## COMPETING INTERESTS

The authors declare no conflict of interest.
